# Low-complexity continuous-variable quantum key distribution with true local oscillator using pilot-assisted frequency locking

**DOI:** 10.1038/s41598-024-61461-0

**Published:** 2024-05-10

**Authors:** Andres Ruiz-Chamorro, Aida Garcia-Callejo, Veronica Fernandez

**Affiliations:** Spanish National Research Council (CSIC), Institute of Physical and Information Technologies (ITEFI), Serrano 144, 28006 Madrid, Spain

**Keywords:** Fibre optics and optical communications, Lasers, LEDs and light sources, Quantum information

## Abstract

In the domain of continuous variable quantum key distribution (CV-QKD), a significant challenge arises in achieving precise frequency synchronization, an issue commonly termed as frequency locking. This involves matching the optical frequencies of both the quantum signal laser and the local oscillator laser for accurate symbol demodulation during the exchange of quantum keys. As such, implementations today still grapple with maintaining precise synchronization between sender and receiver frequencies, occasionally hindering the efficiency and reliability of the information exchange. Addressing this challenge, we present and empirically validate a novel approach to CV-QKD by incorporating a pilot tone-assisted frequency locking algorithm to enhance stability when using a locally generated local oscillator (LLO) at the receiver. The proposed design leverages software-based optimization techniques, thereby eliminating the need for high-speed electronic stabilization devices and achieving efficient performance at typical repetition rates. Specifically, the introduction of the pilot tone algorithm allows us to effectively mitigate phase fluctuations and preserve the integrity of the quantum signals during transmission without resorting to time-multiplexed reference pulses or fast-locking electronics in the lasers. Our results suggest the potential for achieving secure key rates of up to 1 Mb/s over a 50 km single-mode fiber when using these techniques, offering promising insights into the feasibility of high-rate, low-complexity CV-QKD implementations under realistic conditions.

## Introduction

Quantum key distribution (QKD) is a cryptographic method designed to establish a secret key between two remote entities^1–3^. Grounded in the principles of Quantum Mechanics, the distinctive importance of QKD lies both in its immunity to attacks reliant on the computational capabilities of an adversary and in its capacity to discern the presence of potential eavesdroppers within the communication channel, adding a layer of security to the safeguarding of sensitive information.

When translating the logical principles of this technique into a tangible solution, and specifically more so upon considering the physical systems employed for the instantiation of information, QKD broadly classifies into two principal protocol categories as of today: discrete variable (DV) QKD, where information is typically encoded in the polarization states of individual photons^4^, and continuous variable (CV) QKD, where information is encoded in the quadratures of the electromagnetic field. Typically, coherent states generated by a continuous-wave laser are employed as carriers for this encoding process^5^, though squeezed states can also serve this purpose^6^.

While discrete-variable QKD (DV-QKD) boasts a longer distance record of 1000 km, this achievement has relied on superconducting nanowire single-photon detectors (SNSPDs)^7^, so its scalability and cost remain a challenge. Advancements in scalability with the development of chip-based MDI-QKD reaching 31 b/s at 180 km^8^ or chip-based BB84 implementations^9,10^ reaching up to 4.9 kb/s at 251.7 km showcase progress, but still relying on high cost SNSPDs. Conversely, continuous-variable QKD (CV-QKD) achieves shorter distances, with a record of 202 km, using significantly cheaper homodyne detectors^11^. Furthermore, integrated chip technology for CV-QKD holds promise for distances up to 100 km^12^ and key rates of 0.75 Mb/s at 50 km^13^.

Despite the theoretical feasibility of CV-QKD for secure key distribution between two parties, experimental implementations encounter significant challenges. A prominent issue in CV-QKD is the local oscillator problem. In a typical scenario, CV-QKD involves modulating a signal onto a laser at the sender (Alice), transmitting it to the receiver (Bob), and then demodulating it using a signal known as the local oscillator. The local oscillator can either be sent alongside Alice’s signal or generated by another laser at Bob’s setup, the latter being known as the locally-located local oscillator or local-local oscillator^14,15^, which is the implementation employed in our system. However, achieving phase coherence between Bob’s local oscillator and Alice’s laser in such a scheme poses a substantial challenge due to the inherent phase disparity in independent laser sources.

An initial solution to this problem involved multiplexing the data quantum signal and the local oscillator in Alice’s laser to transmit both signals through the channel^16^. However, later studies^17–19^ revealed that this approach had several drawbacks compromising both security and implementation viability, such as the wavelength attack, which allows the eavesdropper to change Bob’s beam splitter outputs by switching the wavelength of Alice’s laser in the channel due to the wavelength-dependent splitting ratio of the beam splitter. The pilot tone^20^ was consequently introduced to solve this problem: Instead of transmitting the local oscillator directly from the transmitter, the approach involves sending a reference pulse (pilot tone) that is multiplexed with the quantum data signal. This reference pulse has a much lower amplitude than the local oscillator, though still greater than the quantum signal.

Traditionally, phase synchronization between the local-local oscillator and the pilot tone has been achieved through control electronics that tune Bob’s laser frequency and phase^21–24^. These electronics synchronize the frequency and phase of Bob’s laser with those of Alice’s laser, using the phase and frequency information extracted from the pilot tone. In this proposal, the electronics of the CV-QKD system are further simplified by performing the demodulation via software-based digital signal processing. The core idea to be detailed in what follows is to allow both Alice’s and Bob’s lasers to fluctuate freely in frequency and phase, performing demodulation despite having mismatched frequency and phase settings. Subsequently, the pilot tone information is used to correct this demodulation via software, mitigating all the errors stemming from phase differences with theoretical perfect precision.

In recent literature, various methodologies have been explored to enhance carrier recovery using machine learning (ML) techniques, demonstrating ML models’ adaptability to varying system conditions without reliance on conventional assumption-based frameworks. These models and techniques significantly improve system parameter estimation and optimization^25^, allowing for transmissions of nearly 100 km by reducing the crosstalk between the data and pilot signals^26^. An alternative approach involves the use of time-multiplexed interleaved pulses or pilots, a strategy that alternates between calibration and data transmission stages. This method commonly employs a phase recovery algorithm during calibration—similar to the feedforward^27^ phase recovery method—to correct phase discrepancies before proceeding with data acquisition^28^. In contrast to the previous, our work introduces a frequency-multiplexed pilot tone combined with a lightweight signal mixing algorithm, for phase-locking within a Gaussian Modulated Local Local Oscillator Continuous Variable Quantum Key Distribution (GM-LLO-CV-QKD) system. The adoption of a frequency-multiplexed pilot tone, as opposed to time-multiplexed alternatives, enables simultaneous transmission of quantum data and synchronization signals. Furthermore, applying this algorithm over ML-based solutions allows for implementation within a software-defined radio, potentially offering better processing times.

Previous studies have demonstrated the potential of LLO-CV-QKD systems employing pilot tone for phase recovery, yielding promising results. Notably, rates of up to 10 Mb/s at 25 km have been achieved through the use of discrete modulation schemes^29^. High distances of up to 100 km have been reached using post-processing phase recovery algorithms employing frequency-multiplexed pilots, akin to our approach^30,31^. The distinctive aspect of our work lies in the employment of a single homodyne detector, contrasting with the use of multiple detectors in the previously referenced work, by integrating the digital signal processing phase recovery method with a low-complexity heterodyne detection framework^32^, and thus significantly simplifying the experimental setup. This simplification resonates with another study that combines a low-complexity approach with a Machine Learning-based carrier recovery algorithm, which achieves remarkably high rates over 100 km distances with an exceedingly straightforward experimental configuration^33^.

## Methods

### Experimental design

**Figure 1:**
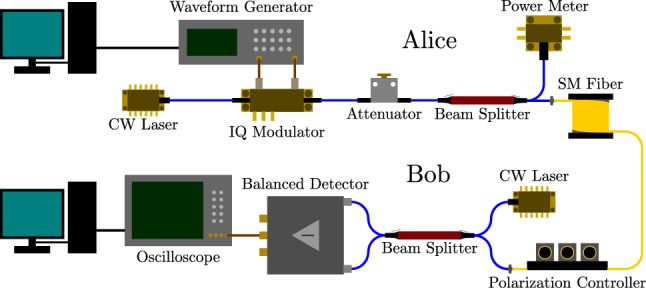
Diagram of the experimental setup. Alice uses an IQ modulator to modulate the amplitude and phase of a continuous wave laser, estimate the modulation variance $V_A$ with a power meter and adjusts an attenuator to send the desired amplitude at the quantum level. Bob corrects the polarization of the incoming signal, combines it with an independent laser known as the locally located local oscillator, before measuring the outputs in a homodyne detector, and performs digital signal processing after acquisition and digitalization.

The experimental system is designed based on low-complexity heterodyne detection techniques in LLO-CV-QKD implementations^32^, as depicted in Fig. 1. This setup features a source laser at Alice and a local oscillator at Bob. Following a Prepare & Measure (P &M) scheme, Alice employs a Quantum Random Number Generator (QRNG) that outputs values according to a Gaussian distribution. Such values will be afterwards processed with a Raised Cosine Filter (RCF), to be ultimately encoded into the quadratures of coherent states by modulating the amplitude and phase of a laser using an IQ modulator, which is maintained at the quadrature transfer point through the use of an external bias controller. Subsequently, the signal is attenuated to the level of a few photons per pulse before being sent to Bob.

Once the signal reaches Bob, it is first corrected with a polarization controller, afterwards mixed with the local oscillator and measured with a homodyne detector, and the electronic output is digitized. Subsequently, the demodulation method based on the pilot tone, to be detailed in the following section, is applied. Ultimately, each of the parties possesses a set of symbols correlated with each other, here denoted $x_i$ at Alice’s end and $y_i$ at Bob’s end, for $i=1,\dots , 2N$, being *N* the total number of symbols transmitted. A portion of these symbols is publicly shared over the classical channel to perform the parameter estimation, while the remaining are to be used as part of the key.

### Pilot-tone assisted frequency locking

**Figure 2:**
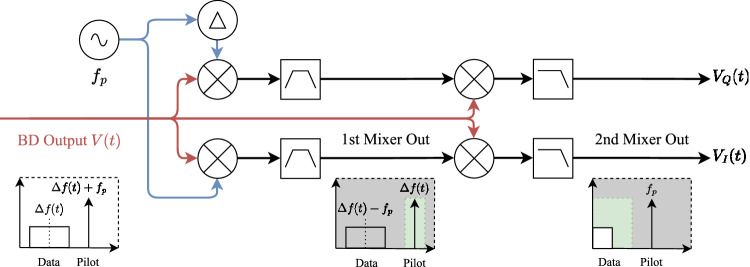
Diagram of the procedure to apply the pilot-tone assisted frequency-Locking method. The pilot-tone mixer consists of multiplying the digitized signal with a sinusoidal signal of exactly the same frequency as the pilot tone. The second mixer (at the right hand side) combines the digitized signal with the previous mixer result after band-pass filtering.

The method here presented provides a novel solution to the problem of the frequency locking and phase stabilization of the transmitter and receiver’s laser signals for CV-QKD implementations using local-local oscillators (LLO). Specifically, the aim is to simplify existing alternatives by leveraging digital signal processing techniques involving the pilot tone to eliminate the need for phase stabilization electronics within the lasers. As such, this technique may be implemented in any heterodyne detection scheme, whether directly or by means of a low-complexity heterodyne detection method^32^, as used in this work. It is important to note that the specific implementation of heterodyne detection is not crucial to the core methodology; variations in the algorithm may occur, but the underlying principle remains consistent across different implementations.

Figure 2 illustrates the demodulation process of the output signal at Bob’s homodyne detector. After digitizing the output signal from the homodyne detector, it undergoes a series of operations. It is firstly mixed with a sinusoidal signal of the same frequency as the reference pilot tone, in order to be subsequently filtered to isolate a sinusoidal signal. The result is then mixed once again with the output signal of the homodyne detector, for Bob to eventually obtain the modulation signals sent by Alice in the first place after low-pass filtering. This procedure exploits the properties of the pilot tone that encapsulates all the information regarding the frequency difference between the lasers. This is so because the received signal *V*(*t*) exhibits a dominant harmonic at $\Delta f(t) + f_p$ in its Fourier spectrum, where $\Delta f(t)$ represents the time-varying frequency difference between the lasers and $f_p$ denotes the frequency of the pilot tone. Initially, *V*(*t*) is mixed with a signal of frequency $f_p$, and after band-pass filtering, a signal of frequency $\Delta f(t)$ is obtained. This signal is then mixed again with *V*(*t*), where the quantum information band is precisely centered at $\Delta f(t)$, effectively yielding the base-band signals $V_I(t)$ and $V_Q(t)$, while the carrier is completely eliminated.

Furthermore, it can be demonstrated that the phase difference between both lasers also cancels out, removing the effects arising from phase differences. To illustrate this quickly, let us assume that the signal *V*(*t*) (for the case of the quadrature *I*, while ignoring signal amplitudes) can be described by an equation of the form:

1 $$\begin{aligned} V(t) \sim \cos \left[ 2\pi \Delta f(t) + \Delta \phi (t)\right] V_I(t) + \cos \left[ 2\pi (\Delta f(t) + f_p)t + \Delta \phi (t)\right] , \end{aligned}$$

where $V_I(t)$ represents the *I* component of the modulation signal sent by Alice, $f_p$ is the pilot tone frequency, $\Delta f(t)$ represents the frequency difference between both lasers, and $\Delta \phi (t)$ stands for the phase difference between the lasers. After multiplying this signal *V*(*t*) by $P(t)=\cos (2\pi f_p t)$ and subsequently filtering it to retain only the component within the $\Delta f(t)$ region with a band-pass filter (BPF), the result is

2 $$\begin{aligned} D(t) \sim \cos \left[ 2\pi \Delta f(t) t + \Delta \phi (t)\right] , \end{aligned}$$

which represents the demodulation signal. Now, this demodulation signal *D*(*t*) is multiplied by *V*(*t*) and filtered using a low-pass filter (LPF) to eliminate all terms with higher frequencies than the symbol frequency (the bandwidth of signals $V_I(t)$ and $V_Q(t)$). The outcome thus obtained is

3 $$\begin{aligned} \text {LPF}[V(t)D(t)] \sim V_I(t). \end{aligned}$$

It can be followed from the above reasoning that the method eliminates the dependence of the demodulation result on both phase differences $\Delta \phi (t)$ and frequency differences $\Delta f(t)$. It is important to note that the correction of drifts takes place upon the complete acquisition of the signal. Therefore, the detector’s bandwidth must exceed the amplitude of random drifts. Once the signal is fully acquired by the detector, this method becomes entirely independent of experimental parameters and imperfections, as shown in the previous equations. This independence extends to factors such as the speed and amplitude of random frequency drifts, as they nullify each other during the algorithm, rendering it a highly effective and lightweight solution for addressing laser drift corrections.

### Secret key rate estimation

Upon completion of a transmission and resolution of the frequency locking issue, the security of the transmission is subsequently assessed. The security assessment relies on a set of techniques commonly known as parameter estimation, which marks the second stage of the GG02 protocol^5,34^, and is widely employed in CV-QKD implementations.

The estimation of the secret key rate in our system, which has been here computed both assuming asymptotic conditions and considering finite-size effects, adheres to the standard procedures employed in conventional CV-QKD systems within the framework of composable security. The security thresholds here computed are robust against collective attacks by Eve on the channel and a realistic model has been adopted. Specifically, we employ the the assumption of the trusted detector model, meaning that the electronic noise $\nu _{\text {el}}$ and detection efficiency $\eta$ are adscribed to Bob’s set up; they are assumed known (characterized), trusted and protected from Eve’s potential tampering.

In such a setting, therefore, the secure key rate $K_{coll}$ of the transmission follows the expression

4 $$\begin{aligned} K_{coll}^{asymp} = f_s (\beta I_{\text {AB}}- \chi _{\text {BE}}), \end{aligned}$$

where $\beta$ represents the reconciliation efficiency, $I_{\text {AB}}$ denotes the mutual information between Alice and Bob, and $\chi _{\text {BE}}$ is the Holevo bound^35^ in a reverse reconciliation scheme. The mutual information can be derived directly from Shannon’s equations^36^ and is expressed as

5 $$\begin{aligned} I_{\text {AB}}= \frac{1}{2}\log _2\frac{V+\chi _{\text {tot}}}{1 + \chi _{\text {tot}}}, \end{aligned}$$

where $V_A$ is the modulation variance and $V=V_A+1$, $\chi _{\text {tot}}=\chi _{\text {ch}}+\chi _{\text {det}}/T$ is the sum of channel noise $\chi _{\text {ch}}=1/T - 1 + \xi$ and detector noise $\chi _{\text {det}}=(1+\nu _{\text {el}})/\eta -1$, with *T* representing channel transmittance and $\xi$ indicating excess noise.

On the other hand, the Holevo bound, serving as an upper constraint to the maximum information accessible to Eve, can be computed in this setting through Von Neumann entropy using the symplectic eigenvalues $\lambda _1$ and $\lambda _2$ of the covariance matrix of the bipartite state shared by Alice and Bob^37^, defined as:

6 $$\begin{aligned} \Sigma _{AB}= \begin{pmatrix} V_A \mathbb {I} &{} \sqrt{T(V^2 - 1)} \sigma _z\\ \sqrt{T(V^2 - 1)} \sigma _z &{} T(V + \chi _{\text {ch}}) \mathbb {I} \end{pmatrix} \end{aligned}$$

where $\mathbb {I}$ and $\sigma _z$ are the identity and the third Pauli matrix respectively; and the eigenvalues $\lambda _3$ and $\lambda _4$ of the covariance matrix of Eve’s mode given Bob’s measurement results (a more detailed review on this topic can be found in Section 7 of^34^). After simplification, the Holevo bound is expressed—in a reverse reconciliation scheme—as

7 $$\begin{aligned} \chi _{\text {BE}} = G\left( \frac{\lambda _1 - 1}{2}\right) + G\left( \frac{\lambda _2 - 1}{2}\right) - G\left( \frac{\lambda _3 - 1}{2}\right) - G\left( \frac{\lambda _4 - 1}{2}\right) , \end{aligned}$$

where $G(x)=(x+1)\log _2(\lambda _i+1) - x \log _2 (x)$. The first two eigenvalues are given by

8 $$\begin{aligned} \lambda _{1,2}=\sqrt{\frac{1}{2} \left[ A \pm \sqrt{A^2-4B}\right] }, \end{aligned}$$

where $A=V^2(1-2T)+2T+T^2(V+\chi _{\text {ch}})^2$ and $B=T^2(V\chi _{\text {ch}}+1)^2$. The other two eigenvalues are given by

9 $$\begin{aligned} \lambda _{3,4} = \sqrt{\frac{1}{2} \left[ C\pm \sqrt{C^2-4D}\right] }, \end{aligned}$$

with $$C=(V\sqrt{B}+T(V+\chi _{\text {ch}}) + A\chi _{\text {det}})/(T(V+\chi _{\text {tot}}))$$, and $$D=(\sqrt{B}(V+\sqrt{B}\chi _{\text {det}}))/(T(V+\chi _{\text {tot}}))$$.

Note that all of the mathematical formalism in this section is applicable to the homodyne detection scenario. In the case of heterodyne detection, while the mutual information from Eq. (5) would double, the detector noise term $\chi _{\text {det}}$ would also increase to $2\chi _{\text {det}} + 1$^34^.

## Results

### Implementation of the pilot-tone assisted frequency locking algorithm

In what follows, we empirically validate the effectiveness of the pilot tone-based frequency locking algorithm presented. As detailed in Section “Methods”, the algorithm’s concept involves for Bob to down-convert the component of the received signal that corresponds to the pilot tone. Such is subsequently filtered to obtain a signal that precisely represents the frequency and phase of the modulation band carrier at all times. This technique ensures demodulation free from any imperfections that may be introduced by an active frequency control loop operating on the laser.

**Figure 3:**
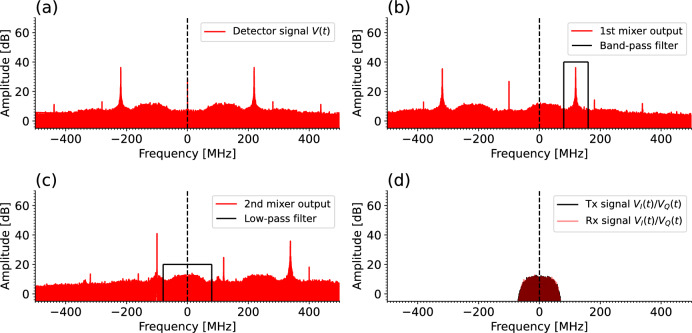
Frequency spectrum of the successive phases of the frequency-locking algorithm using experimental signals. (**a**) shows the output of the homodyne detector, which is obtained from the experimental setup and digitized. (**b**) shows the spectrum of the signal generated numerically at the output of the first mixer, including the cutoff frequencies of the band-pass filter employed. (**c**) shows the output of the second mixer and the cutoff frequencies of the low-pass filter. (**d**) shows the original transmitted signal and the final received signal obtained after the low-pass filter, which is permanently centered at 0 Hz, regardless of the carrier frequency drift in the lasers.

In Fig. 3a, the Fourier spectrum of the signal at the output of the homodyne detector upon reception is displayed. Figure 3b shows the results of the previous multiplied by a sinusoidal wave of frequency $f_p$ matching the pilot tone, and filtered with a band-pass filter with cutoff frequencies in the approximate expected region for $\Delta f$. Figure 3c displays the result of multiplying the received signal by this previously filtered signal, and the final outcome after applying a low-pass filter to remove the pilot tone is displayed in Figure 3d.

**Figure 4:**
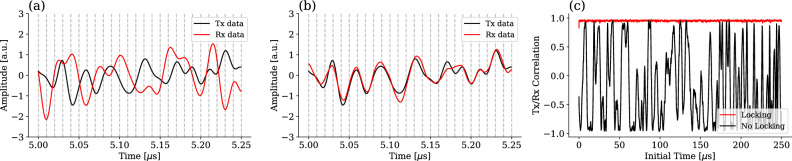
Time-domain plots of the transmitted (Tx) and received (Rx) modulation signals, containing the quantum data symbols following a Gaussian distribution. The transmitted signal consists on the Gaussian symbols processed with a Raised Cosine Filter, while the received signal is the obtained signal after all the demodulation. (**a**) shows the result without using any frequency-locking technique and (**b**) using the proposed frequency-locking method. (**c**) Shows the correlation between chunks of 100 sampled symbols from the transmitted and received modulation signals, starting from a given initial time.

Equivalently, Fig. 4a shows the transmitted and received modulating signals in the time domain for a portion of the data block. Note that the data in Figs. 3 and 4 have been obtained from experimental measurements of a real transmission of 5 km, using the CV-QKD setup, but using bright pulses at a level of more than 100 photons per pulse for visualization purposes. Otherwise, the quantum signal would be at the noise level and the effectiveness of the frequency locking algorithm would not be discerned as easily. Also note that both Figs. 3 and 4 represent only the In-Phase component of the phase space, since the Q quadrature representation is equivalent.

Furthermore, it’s worth highlighting that phase and frequency coherence is maintained during the time that the entire signal lasts, as shown in Fig. 4c. Here it can be observed that when no locking algorithm is implemented, and the low-complexity homodyne demodulation is carried out directly using a fixed down-conversion frequency, which can be obtained measuring the pilot tone component frequency and subtracting $f_p$, the correlation randomly fluctuates between – 1 and 1 as the frequency of both lasers freely drift during the entire signal duration. In contrast, implementing the frequency-locking algorithm results in a consistent correlation over time, ensuring the signal is correctly demodulated, as discussed in Section “Methods”.

### Experimental demonstration of the CV-QKD system

In what follows, we verify the efficacy of the method by using it to perform an experimental CV-QKD transmission. Following demodulation, the security of the transmission will be assessed. To achieve this, the channel noise parameters are estimated, namely the transmittance and the excess noise, based on the relationship between the transmitted symbols, *x*, and the received symbols, *y*. In the finite-size regime the previous can be achieved by computing the maximum likelihood estimators for the channel transmittance, ${\hat{T}}$, and excess noise, ${\hat{\xi }}$. The estimator for the channel transmittance is defined by

10 $$\begin{aligned} {\hat{T}} = \frac{1}{\eta }\left( \frac{\text {Cov}(x,y)}{\text {Var}(x)}\right) ^2. \end{aligned}$$

This estimator represents the covariance between *x* and *y* divided by the variance of *x*, scaled by the detection efficiency $\eta$. On the other hand, the excess noise $\xi$ is estimated as

11 $$\begin{aligned} {\hat{\xi }} = \frac{1}{\eta {\hat{T}}} \left[ \frac{1}{2N}\sum _{i=1}^{2N}\left( y_i-\sqrt{\eta {\hat{T}}}x_i\right) ^2 - 1 - \nu _{\text {el}}\right] , \end{aligned}$$

where *N* stands for the total number of transmitted and received symbols, and $\nu _{\text {el}}$ represents the electronic noise assumed by Bob in the trusted noise model as described in^38^. By estimating transmittance and excess noise in this manner, and by calculating the secret key rate as described in Section “Methods”, we conduct both simulations and experimental transmissions for several increasing channel lengths. The parameters with which the setup was configured for the experimental transmission are listed in Table 1.

**Table 1:** Values of the different parameters used in the experimental transmission and in the secret key rate estimation.

Parameter	Value
Number of symbols	$10^6$
Symbol frequency ($f_s$)	100 MHz
Pilot tone frequency ($f_p$)	100 MHz
DAC sampling rate	5 GS/s
ADC sampling rate	2 GS/s
Pilot tone/modulation band	30 dB
Laser frequency	193.5 THz
Local oscillator power	10 dBm
Optical fiber attenuation	0.2 dB/km
Detector noise equivalent power	4.5 pW / $\sqrt{Hz}$
Detector gain	5 V/mW
Detector bandwidth	400 MHz
IQ modulator half-wave voltage ($V_\pi$)	$\sim$ 4 V
Modulation variance ($V_A$)	$\sim$ 5 SNU
Electronic noise ($\nu _{\text {el}}$)	0.1084 SNU
Detector efficiency ($\eta$)	0.55
Reconciliation efficiency ($\beta$)	0.95
Confidence intervals ($\epsilon _\text {PE}$)	$10^{-10}$

The values used for the electronic noise, and detector efficiency in the security analysis were previously experimentally characterized.

The experiment, based on the setup introduced in Fig. 1, consists on sending $10^6$ symbols following a normal distribution, all of which are subsequently filtered out with a RCF, and encoded in the I and Q quadratures of a 1550 nm C-Band CW tunable laser using an IQ modulator controlled by an arbitrary waveform generator. Afterwards, the signal is attenuated to a modulation variance of around 5 SNU (2.5 photons per pulse), and the power is continuously monitored to keep track of such modulation variance in each transmission. The signal is then sent to three different channels of 5 km, 25 km and 50 km, all three of which are Single Mode Fiber (SMF) reels. The polarization at the output is corrected using a manual polarization controller to maximize the amplitude of the beam-splitter interference with Bob’s laser (which is the same model as that of Alice’s). After the interference, the outputs are measured in a balanced detector, whose subsequent output is acquired using a digital oscilloscope.

In each of the three experiments, the shot noise is estimated just before each transmission, by measuring first the variance of the local oscillator $N_0$ (by cutting off the channel entrance), and secondly the variance of the electronic noise $\nu _{\text {el}}$ (by switching off Bob’s laser). The shot noise unit conversion is then given by $N_0-\nu _{\text {el}}$.

Once the acquisition is finalised, the signal is processed with the frequency-locking algorithm, and afterwards sampled to retrieve $10^6$ symbols. Half of them are to be used as the key and the remaining for parameter estimation. The values obtained using the second group are listed in Table 2 below.

**Table 2:** Worst-case estimation of the channel noise parameters and secret key rate for different channel lengths.

*L* (km)	${\hat{T}}$	${\hat{\xi }}$ (SNU)	*K* (b/sym)
5	0.83	0.091	0.09180
25	0.30	0.092	0.01030
50	0.09	0.091	0.00072

**Figure 5:**
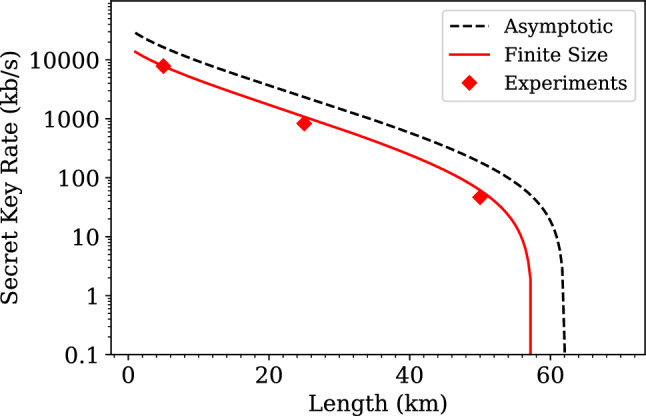
Experimental results for 5 km, 25 km, and 50 km and simulations for different distances. The finite-size simulations use $10^6$ symbol key blocks and take the worst-case scenario estimator for the transmittance and the excess noise, which effectively outputs a tighter estimation of the secure key rate. Note that each point the finite-size simulation curve is the average of multiple simulations for a given distance, since each simulation run might produce slightly different results.

The results of the previous secret key rate estimations are also displayed in Fig. 5, along with those derived from simulations. In detail, such simulations have been carried out both in the asymptotic regime, for reference, and taking into account finite-size effects.

In detail, to perform simulations in the asymptotic limit, the pre-computed transmittance values for each distance have been used, according to the analytic expression $T=10^{-\alpha L /10}$ for distance *L* and channel attenuation per km $\alpha$. Similarly, a $\xi =0.09$ SNU value for the excess noise has been used.

For simulations considering finite-size effects, we follow the methods described in^39^. We generate blocks of simulated symbols of the same size as those sent in the real transmission, of the form $x_{i}\in {\mathcal {N}}(0, V_A)$ for $i=1, \ldots ,2N$ following a normal distribution. These elements undergo attenuation due to efficiency and transmittance, as well as noise, evaluated according to the linear model:

12 $$\begin{aligned} y = \sqrt{\eta T} x + z, \end{aligned}$$

where *z* represents Gaussian noise with zero mean and variance $1+\xi +\nu _{\text {el}}$, with $\nu _{\text {el}}$ being the experimentally characterized electronic noise and $\xi$ being the average measured excess noise in the experiments.

Afterwards, for an error probability of $\epsilon _\text {PE}= 10^{-10}$ in the parameter estimation stage, confidence intervals^40^ for the transmittance $\Delta T$ and the excess noise $\Delta \xi$ are defined using the inverse error function $z_\text {PE}=\sqrt{2} \textrm{erf}^{-1}(1-\epsilon _\text {PE})$ as

13 $$\begin{aligned} \Delta T = z_\text {PE}\sqrt{\frac{\sigma ^2}{2N V_A}}, \quad \Delta \xi = z_\text {PE}\sqrt{\frac{1}{N}} (\sigma ^2-1 - \nu _{\text {el}}), \end{aligned}$$

where $\sigma ^2=\eta T \xi + 1 + \nu _{\text {el}}$. The worst-case estimator for the transmittance is given by

14 $$\begin{aligned} T^* = \frac{1}{\eta }\left( \sqrt{\eta T} - \Delta T\right) ^2, \end{aligned}$$

while the worst-case estimator for the excess noise is defined as

15 $$\begin{aligned} \xi ^* = \frac{1}{\eta T} (\sigma ^2 + \Delta \xi - 1 - \nu _{\text {el}}). \end{aligned}$$

Using these two estimators for the main channel noise parameters, we then estimate the secret key rate according to the expression introduced in Section “Secret key rate estimation”, noting that its magnitude is indeed scaled when taking into consideration the finite size effects^39^,

16 $$\begin{aligned} K^{fin}_{coll} = f_s \frac{N-m}{N} (\beta I_{\text {AB}}- \chi _{\text {BE}}), \end{aligned}$$

where *N* is the length of the total sent and received symbols, and *m* is the number of symbols used for parameter estimation.

### Discussion

Our results, on one hand, confirm the effectiveness of the pilot-tone-based frequency-locking algorithm in maintaining phase and frequency coherence over the entire signal. This underscores the feasibility of employing this algorithm to ensure robust and stable CV-QKD implementations.

On the other hand, the secure key rate results, as demonstrated, not only showcase the practical viability of our CV-QKD system under varying channel distances but also affirm that the system’s performance in an actual transmission, evaluated through the secret key rate, aligns with the projected tendency from theoretical simulations. These simulations, conducted within a standard security framework, accurately predict the system’s behavior within the finite-size regime, effectively adhering to the expected curve. Furthermore, the observed key rates adhere to the upper threshold set by asymptotic regime estimations, further emphasizing the robustness and consistency of our system’s performance across varying operational conditions.

## Conclusions

In this study, we have presented a pivotal advancement for the practical implementation of continuous-variable quantum key distribution (CV-QKD) systems within the broader context of quantum key distribution (QKD). Employing a comprehensive approach, we have particularly focused on enhancing security and practicality in real-world quantum communication scenarios.

Our research initiated with a thorough examination of a pilot-tone-assisted frequency-locking algorithm designed to ensure phase and frequency coherence in CV-QKD systems. Through a combination of experimental and simulated validations, we demonstrate the algorithm’s efficacy, highlighting its potential to streamline active frequency control loops on lasers and affirming the practical viability of the implementation across various channel distances. These findings underscore the adaptability and resilience of our approach, representing a significant stride towards practical quantum communication solutions.

In conclusion, this approach enhances flexibility and accuracy while reducing the complexity of hardware-based synchronization methods. Our study contributes to the advancement of cost-effective and low-complexity continuous-variable quantum key distribution systems, fostering the development of faster and more secure QKD systems. This result paves the way for the development of secure and efficient quantum communication systems in real-world scenarios.

## Data Availability

The datasets generated and/or analysed during the current study are available in the *figshare* repository, at https://doi.org/10.6084/m9.figshare.25222115.
